# A novel *MAP3K20* mutation causing centronuclear myopathy-6 with fiber-type disproportion in a Pakistani family

**DOI:** 10.1038/s10038-022-01085-2

**Published:** 2022-10-11

**Authors:** Ilyas Ahmad, Ayaz Khan, Hafiza Noor Ul Ayan, Birgit Budde, Janine Altmüller, Asad Aslam Korejo, Gudrun Nürnberg, Holger Thiele, Muhmmad Tariq, Peter Nürnberg, Jeanette Erdmann

**Affiliations:** 1 Institute for Cardiogenetics, University of Lübeck, Lübeck, Germany; 2 DZHK (German Research Center for Cardiovascular Research), Partner Site Hamburg/Lübeck/Kiel, 23562 Lübeck, Germany; 3 University Heart Center Lübeck, 23562 Lübeck, Germany; 4 Cologne Center for Genomics (CCG), Faculty of Medicine, University Hospital Cologne, University of Cologne, 50931 Cologne, Germany; 5 National Institute for Biotechnology and Genetic Engineering College, Pakistan Institute of Engineering and Applied Sciences (NIBGE-C, PIEAS), Faisalabad, 38000 Pakistan; 6 National Institute of Cardiovascular Disease, Karachi, 75510 Pakistan; 7 Center for Molecular Medicine Cologne (CMMC), Faculty of Medicine, University Hospital Cologne, University of Cologne, 50931 Cologne, Germany

**Keywords:** Genetics research, Medical genetics

## To the Editor:

Centronuclear myopathy-6 with fiber disproportion (CNM6 [MIM: 617760]) is a rare disorder caused by pathogenic variants in the gene encoding mitogen-activated protein triple kinase 20 (MAP3K20), also known as ZAK. MAP3K20-related myopathy (MAP3K20-RM) was first described in 2017 in three families of different ethnic backgrounds with overlapping phenotypes. To date, only three causative mutations have been described in six patients. The disease typically runs in families and is inherited in an autosomal recessive manner. It manifests as slowly progressive muscle weakness that begins in infancy or early childhood. Affected individuals are hypotonic at birth, but all show delayed motor development and walking difficulties due to muscle weakness, which mainly affects the proximal upper limb and distal lower limbs. Additional features are winged scapulas, hyperlordosis, scoliosis, and mildly decreased respiratory vital capacity; a waddling gait, quadriceps, and calf hypertrophy have also been reported in some patients. None of the patients had facial weakness, dysphagia, or cardiac involvement. The phenotype and muscle biopsy abnormalities are variable; however, centralized nuclei and disproportionate fiber size have been the common histopathological features [1].

**Fig. 1:**
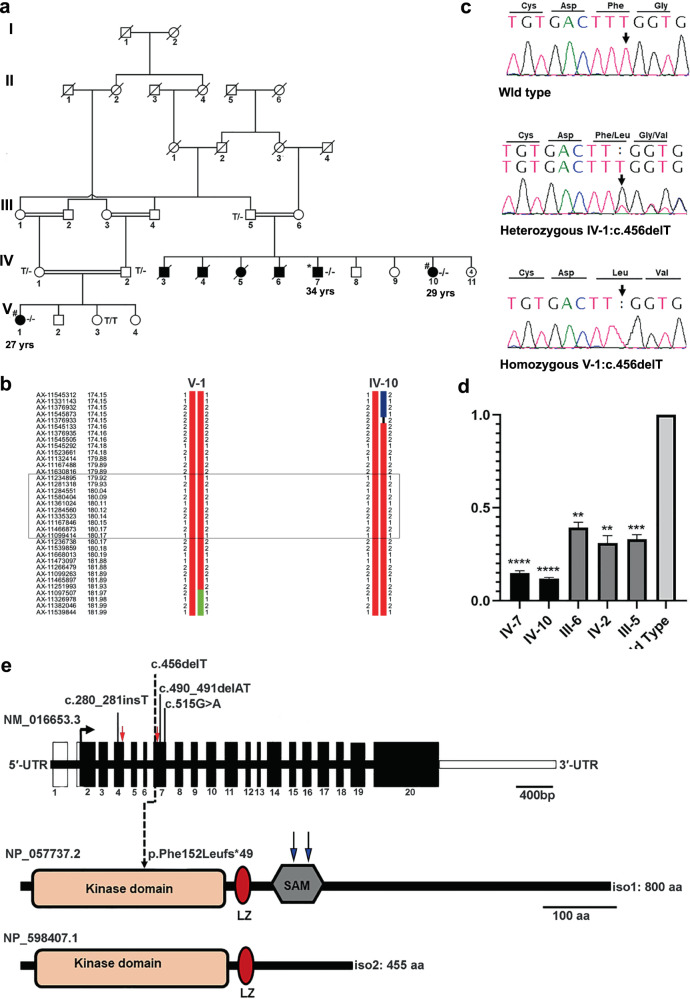
Identification of a homozygous mutation c.456delT in *MAP3K20*. **a** A large consanguineous family with congenital myopathy. Filled symbols represent affected individuals; open symbols represent unaffected individuals; squares denote males and circles denote females; symbols with a diagonal line denote deceased individuals. The genotype of the *MAP3K20* mutation is indicated below each examined member: TT (wildtype), T/– (heterozygous), and –/– (homozygous mutant). An asterisk (*) indicates the individual for whom exome sequencing was performed. A hash (#) designates individuals for whom genotyping was performed. The number indicates the current age of each living affected individual. **b** Recombination events in individuals V:I and IV:10 delimit the region of interest between flanking markers AX-11545873 (174.15 cM, 169757541 bp) and AX-11097507 (181.96 cM, 175417011 bp). Physical positions refer to human genome build GRCh37.p13 (hg19). The *MAP3K20* location is boxed. **c** Representative sequence chromatograms showing the normal sequence, c.456delT, in heterozygous and homozygous individuals. The vertical arrow indicates the site of the mutation. **d** Quantitative real-time PCR of MAP3K20. Levels of functional MAP3K20 are reduced to 12–15% of the normal level in patients with the c.456delT mutation. RNA was extracted from the patients, heterozygous carriers, and a healthy control. qPCR reactions were run in triplicate in three independent experiments (*n* = 3). Data are expressed as the mean ± SEM (****P < 0.001, ***P < 0.001, and **P < 0.01; compared with MAP3K20 wildtype). All data were normally distributed (Shapiro–Wilk test). *P* values were calculated using an unpaired, parametric, one-tailed Student’s *t*-test, assuming unequal SDs. **e** Schematic representation for gene and protein domain structure of the human MAP3K20. The red arrow marks the region for primers in exons 4 and 7 for RT-PCR. Previously reported mutations ([c.280_281insT p.Asn95*]; [c.490_491delAT p.Met164fs*24 [c.515G>A; p.Trp172*]) associated with CNM6 are shown in black [1], whereas the novel mutation (c.456delT, p.Phe152Leufs*49) identified in this study is shown by a dotted line. Blue arrows indicate the positions of mutations associated with SHFM described by Spielmann et al. [6]. The MAP3K20 protein comprises a kinase domain (16–277 aa), a leucine-zipper (LZ; 287–308 aa), and a sterile-alpha motif (SAM) domain (336–410 aa) in isoform 1. Two isoforms (Iso 1 and Iso 2) are known, and these differ in their C terminus

Here, we report the molecular investigation of a consanguineous Pakistani family presenting with slowly progressive congenital myopathy, with a preferred quadrupedal gait. The family lives in a rural area of Sindh province, Pakistan. Four males and three females were affected, distributed in two loops (Fig. 1a). Four of these affected individuals (V:3, V:4, V:5, and V:6) died in early childhood; however, the exact cause of death could not be confirmed. The study was approved by the Institutional Research Ethics Committee of the National Institute for Biotechnology and Genetic Engineering, Faisalabad, and all the participating individuals provided informed consent. All patients were floppy at birth and showed delayed motor development. At 4–6 years of age, they complained of pain in the legs and experienced difficulty standing from a sitting position. They presented with slow and progressive muscle weakness in the upper and lower limbs. They were unable to climb stairs without support. Their faces were slightly elongated, but had no symptoms of weakness in facial muscles. They also had mild scoliosis and calf hypertrophy at 4–6 years. At the last assessment, all patients had hyperlordosis and winged scapula. They still complain of fatigability and diurnal cramps. In addition, we noticed that these patients are no longer able to stand or walk and are used to sliding in a sitting position to achieve limited locomotion; to move over relatively longer distances, they prefer quadrupedal gait. All patients were born to healthy consanguineous couples.

We performed whole-exome sequencing on DNA from individual V:7 (Fig. 1a) to identify the causal variant in this family. Since the parents were consanguineous, we focused on tracts of autozygosity (≥5 Mb) detected by runs of homozygous single nucleotide variants. Exome sequencing and the filtering process have been described in detail elsewhere [2, 3]. These methods were complemented by SNP-array-based homozygosity mapping of two affected individuals (V:10, VI:1) using the Axiom® Genome-Wide CEU 1 Array (Affymetrix, Santa Clara, CA) (Fig. 1b). Filtered exome variants and regions of homozygosity were superimposed, assuming autosomal recessive inheritance (inferred from the family structure and known consanguinity), full penetrance, and an allelic frequency of 0.0001 [3]. This led to the identification of a novel homozygous single nucleotide deletion (c.456delT) in exon 7 of the *MAP3K20*. The mutation generates a frameshift variant that leads to premature termination of translation (p.Phe152Leufs*49). Sanger sequencing of the available individuals validated the variant and confirmed its segregation in the family (Fig. 1c). It has not been previously reported and absent from all public databases (HGMD; gnomAD; dbSNP). Next, we performed quantitative RT-PCR to investigate the effects of this mutation on transcript levels. As anticipated, there was a marked reduction in *MAP3K20* transcripts in all patients when compared with a healthy control, which is consistent with nonsense-mediated decay (NMD) (Fig. 1d).

MAP3K20 belongs to the mitogen-activated protein triple kinase (MAP3K) family, which is implicated in signal transduction. It comprises an N-terminal kinase domain encoded by exons 2–9, followed by two dimerization domains: a leucine-zipper motif and a sterile-alpha motif (SAM) (Fig. 1e). The MAP3K20 gene produces two protein isoforms; the longer isoform 1 (iso1:800 amino acids) is expressed in different tissues, while the shorter isoform 2 (iso2:455 amino acids) is predominantly expressed in skeletal muscle and the heart [1, 4, 5]. These isoforms are identical except that isoform 2 differs in the 3’UTR and does not encode the SAM domain. Interestingly, mutations in both isoforms cause different disease phenotypes. The mutation in isoform 1, present in the SAM domain, was previously tied with split-foot malformation with mesoaxial polydactyly [MIM: 616890], whereas isoform 2 mutations were associated with CNM6, affecting the kinase domain of MAPK320 protein [1, 6]. Therefore, we hypothesize that the reduction in MAP3K20 mRNA caused by p.Phe152Leufs*49 is restricted to muscle cells.

Our patients share most of their clinical symptoms with previously reported patients homozygous for mutations in the kinase domain of MAP3K20. However, no previous study reported quadrupedal locomotion in such patients. Even the Pakistani patients reported by Vasli et al. did not exhibit this clinical feature [1].

In conclusion, we identified a novel frameshift pathogenic variant of *MAP3K20* causing congenital myopathy, thereby extending the genotypic and phenotypic spectrum of this disease. Understanding the full pathological spectrum of *MAP3K20* mutations necessitates investigating further families with myopathies.

## References

[1] Vasli N, Harris E, Karamchandani J, Bareke E, Majewski J, Romero NB (2017). Recessive mutations in the kinase ZAK cause a congenital myopathy with fibre type disproportion. Brain..

[2] Ramzan S, Tennstedt S, Tariq M, Khan S, Noor Ul Ayan H, Ali A, et al. A novel missense mutation in TNNI3K causes recessively inherited cardiac conduction disease in a consanguineous Pakistani family. Genes (Basel). 2021;12:1282. 10.3390/genes12081282 PMC8395014 34440456

[3] Ahmad I, Baig SM, Abdulkareem AR, Hussain MS, Sur I, Toliat MR (2017). Genetic heterogeneity in Pakistani microcephaly families revisited. Clin Genet.

[4] Liu TC, Huang CJ, Chu YC, Wei CC, Chou CC, Chou MY (2000). Cloning and expression of ZAK, a mixed lineage kinase-like protein containing a leucine-zipper and a sterile-alpha motif. Biochem Biophys Res Commun.

[5] Bloem LJ, Pickard TR, Acton S, Donoghue M, Beavis RC, Knierman MD (2001). Tissue distribution and functional expression of a cDNA encoding a novel mixed lineage kinase. J Mol Cell Cardiol.

[6] Spielmann M, Kakar N, Tayebi N, Leettola C, Nurnberg G, Sowada N (2016). Exome sequencing and CRISPR/Cas genome editing identify mutations of ZAK as a cause of limb defects in humans and mice. Genome Res.

